# Gaze Tracking and Point Estimation Using Low-Cost Head-Mounted Devices †

**DOI:** 10.3390/s20071917

**Published:** 2020-03-30

**Authors:** Ko-Feng Lee, Yen-Lin Chen, Chao-Wei Yu, Kai-Yi Chin, Chen-Han Wu

**Affiliations:** 1 Department of Computer Science and Information Engineering, National Taipei University of Technology, Taipei 10608, Taiwan; t104599001@ntut.edu.tw (K.-F.L.); david741002@gmail.com (C.-W.Y.); kookyrational@hotmail.com (C.-H.W.); 2 Department of Digital Humanities and Information Applications, Aletheia University, New Taipei City 25103, Taiwan; au0292@mail.au.edu.tw

**Keywords:** eye tracking, gaze estimation, head-mounted, wearable devices, mobile devices

## Abstract

In this study, a head-mounted device was developed to track the gaze of the eyes and estimate the gaze point on the user’s visual plane. To provide a cost-effective vision tracking solution, this head-mounted device is combined with a sized endoscope camera, infrared light, and mobile phone; the devices are also implemented via 3D printing to reduce costs. Based on the proposed image pre-processing techniques, the system can efficiently extract and estimate the pupil ellipse from the camera module. A 3D eye model was also developed to effectively locate eye gaze points from extracted eye images. In the experimental results, average accuracy, precision, and recall rates of the proposed system can achieve an average of over 97%, which can demonstrate the efficiency of the proposed system. This study can be widely used in the Internet of Things, virtual reality, assistive devices, and human-computer interaction applications.

## 1. Introduction

With the development of technology, people can easily obtain knowledge. Most of the information reception in humans is dependent on vision. Visual information can be applied for detecting user behaviors. The pupil movement reflects human gaze. Eyes are one of the primary sensory organs and can distinguish between features of light, distance, and color. Gaze tracking and point estimation are crucial topics in human-computer interaction research fields.

The state of mental activity of a person can be determined by analyzing the trajectories of pupil movements. Similarly, several human behavior traits can be understood using eye trackers. For example, Wang et al. [1] presented a controller for visual tracking control of a robot manipulator. In this controller auto setting camera, the intrinsic and extrinsic parameters are uncalibrated in three-dimensional (3D) general motion. Andaluz et al. [2] presented an image-based dynamic visual feedback control for mobile manipulators to solve the target tracking problem in three-dimensional (3D) workspaces.

An eye tracker determines the trajectories of pupil movement. In recent years, gaze tracking was introduced for related research in neuroscience, psychology, education, marketing, internet of things, and advertisement analysis. Furthermore, eye trackers can be used to devise communication tools for physically disabled people. People with physical disability communicate through eye movements and blinking. Therefore, eye trackers can be used to facilitate communication with the outside world [3]. Traditional eye trackers that have been developed for specific applications are expensive. To overcome this issue, a low-cost wearable device that can function as an accurate eye tracker is proposed in the study.

Wearable devices have gradually become popular over the last few years. These devices can assist the user in games and enhance education. The gaze point and its movement trajectory can provide a quick and intuitive secondary indicator for the human–machine interface and optimize information for improved user experience [4]. For example, John and Sunny [5] determined the ability of students to solve problems by using eye trackers. Copeland and Gedeon [6] used eye trackers to determine the reading preferences of students. Lin et al. [7] used eye trackers to investigate the difference in cognition of students.

Eye-tracking detection algorithms have been classified as model-based [8] and feature-based methods [9]. Typically, in model-based methods, the vision is matched with preset shapes and model formulas, and subsequently, the optimal solution among the limited candidates is determined through voting or classification. Feature-based methods typically use the features of the eyes. Compared with model-based methods, feature-based methods may require a considerably smaller code that can be used in real time. In this study, the camera that captures the movement of the pupils was connected to the computer through Internet. Thus, this study extended our previous implementation [10] and propose a highly stable and accurate feature-based method developed to track gaze and devise a pupil elliptical information extraction method to be used in low-cost head-mounted applications. Moreover, we implemented the proposed algorithms on a low-cost wearable device to provide an efficient wearable eye-tracking solution.

## 2. Related Works

**Figure 1:**
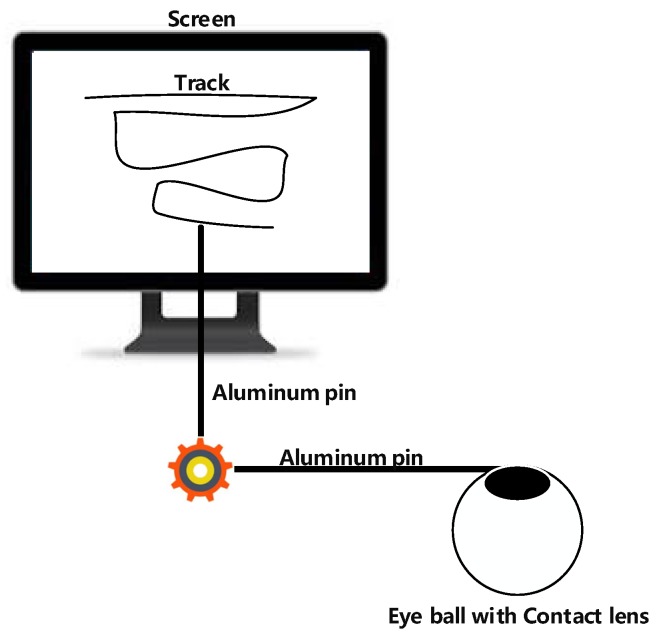
Invasive eye tracker [11].

Huey [11] developed the first invasive eye tracker in the 20th century (Figure 1). In 1935, Buswell et al. [12] developed the first noninvasive eye tracker. In this eye tracker, a beam of light was sent into the eye of the user, and the reflection from the eye was recorded. This eye tracker was more convenient than invasive eye trackers. Therefore, most current eye trackers are noninvasive and can be applied in various fields.

Several studies have determined some methods of developing eye trackers. For example, Kao et al. [8] proposed a model-based eye tracker in which an eye model was defined and the position of the iris on the image in the model was tracked to determine the fixation. Świrski and Dodgson [9] proposed a shape-based eye tracker in which an image of the pupil is used to extract ellipse and reflection projection ellipse information to develop the eye model. However, this method is not suitable for computationally intensive tracking. Sirohey et al. [13] used the edge color information of the pupil and iris to obtain the threshold value from the color information around the eye. Yang et al. used high-quality images and nonlinear filters to identify suitable candidate points for formalizing information of the iris for accurate detection [14]. Based on shapes and additional light sources, Sigut and Sidha [15] proposed a method for supporting head movement to combine the bright spots reflected on the iris. However, these eye trackers are too expensive to be popular for most users.

Some researchers have developed cheap eye trackers. For example, Lee et al. proposed a method for a portable device that involved installing multiple sets of LED lights on the device. Jen et al. [16] proposed a novel wearable eye-gaze tracking system with a single webcam that was mounted on glasses. Dobeš et al. [17] developed a successful method of eye and eyelid localization on a computer by using a modified Hough transform. Lee et al. [18] proposed a novel wearable eye-gaze tracking system with a webcam and used a screen to perform sight verification.

In this study, a low-cost head-mounted device was combined with a feature-based method to achieve a computationally efficient solution with an accurately calibrated device. This solution can provide a solution of low-cost and highly accurate wearable eye tracker.

## 3. Proposed Device

The infrared eye tracker was used in a low-cost head-mounted device. In this study, we aim to reduce the cost of the eye tracker. Therefore, the circuit and mechanism were printed using 3D printing technology. A camera, infrared light-emitting element, power supply, filter, and head-mounted device were incorporated in the proposed device.

### 3.1. Camera Module

**Figure 2:**
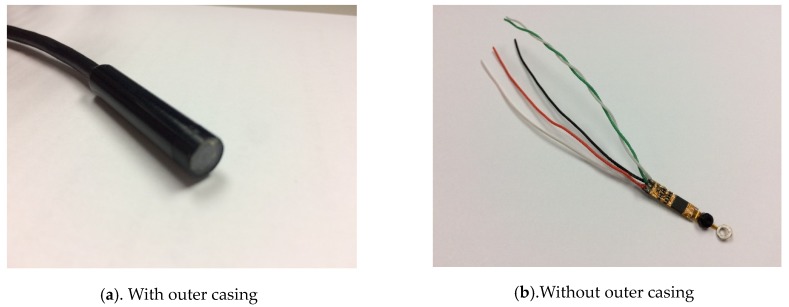
The proposed camera modules.

As depicted in Figure 2a,b, an endoscope camera was connected to a mobile phone by using a micro USB interface. Such endoscope cameras can be connected to both computers and Android phones. These endoscope cameras can be used in low-light environments. The focal length of the camera was fixed at 4 cm, and protocol support was provided for connecting Android mobile phones. The endoscope camera can support resolutions of 320 × 240, 640 × 480, 1280 × 720, and 1600 × 1200.

### 3.2. Infrared Light-Emitting Element and Power Supply

The infrared light-emitting element was an infrared LED, OSRAM SFH 4050-Z, which is a high-power infrared light-emitting element developed by OSRAM Opto Semiconductors. The OSRAM Opto Semiconductors is one of the globally leading brands in the light industry. OSRAM covers the key technologies of the light market as well as the central applications ranging from visible light for illumination to invisible light, e.g., for sensor technology. To move forward OSRAM broadened its portfolio through acquisitions complementing the competencies of the company in specific technology and application fields [19]. The voltage rating was 2.4 V and the current rating was 100 mA. The emission power was 7 mW/sr, and the emission wavelength was 860 nm.

To maintain the brightness and power of the infrared light element, a power supply constant-current module was additionally designed. An LM-317 adjustable linear voltage regulator IC [20] with an SMD (Surface Mount Device [21]) resistor was used in the module to stabilize the output current to 100 mA and power to 140 mW.

### 3.3. Camera Module

**Figure 3:**
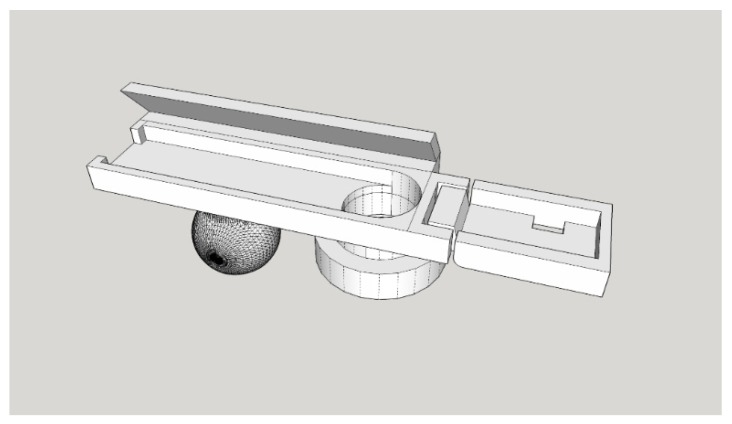
Three-dimensional diagram of the camera module housing.

**Figure 4:**
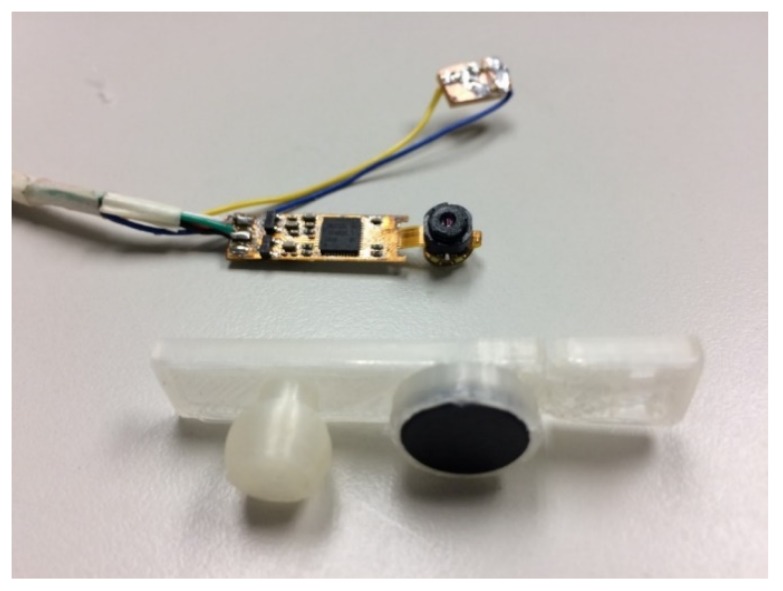
Camera module housing.

As shown in Figure 3, this study used 3D printer to make the module housing. The camera module housing illustrated in Figure 4 was used to house the camera, infrared light-emitting element, and filter. The camera module housing was constructed using a 3D printer.

In the housing, the electronic components were placed in a fixed position. The built-in joint was constructed at the location of the infrared light-emitting element. The housing can be rotated directly to adjust the infrared light illumination angle. An extension plate on the back of the casing can be attached to the head-mounted device, as depicted in Figure 4.

### 3.4. Low-Cost Head-Mounted Device

**Figure 5:**
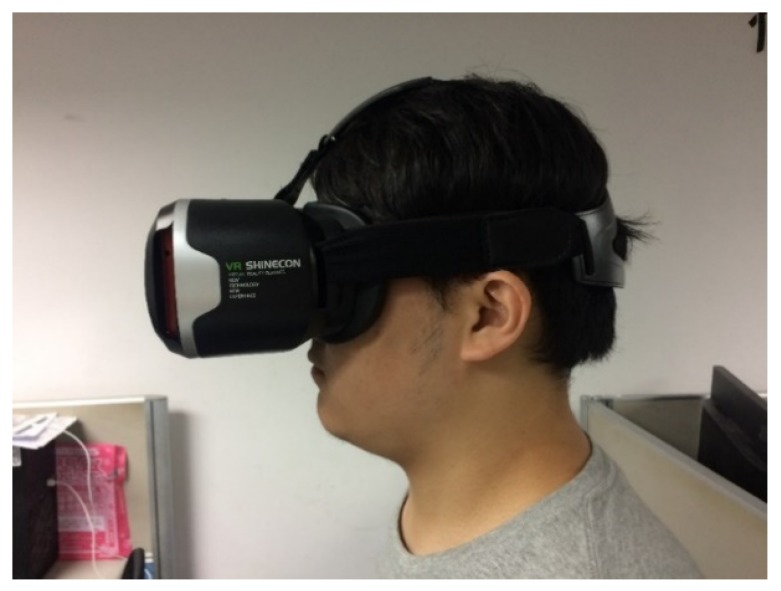
The head-mounted device.

Figure 5 shows the proposed head-mounted device. The eye tracker in the head-mounted device used a mechanism to adjust the distance between the screen and the eyes and did not require chin support for correction and mapping. So that the light source environment can be adjusted to be simple and clean.

**Figure 6:**
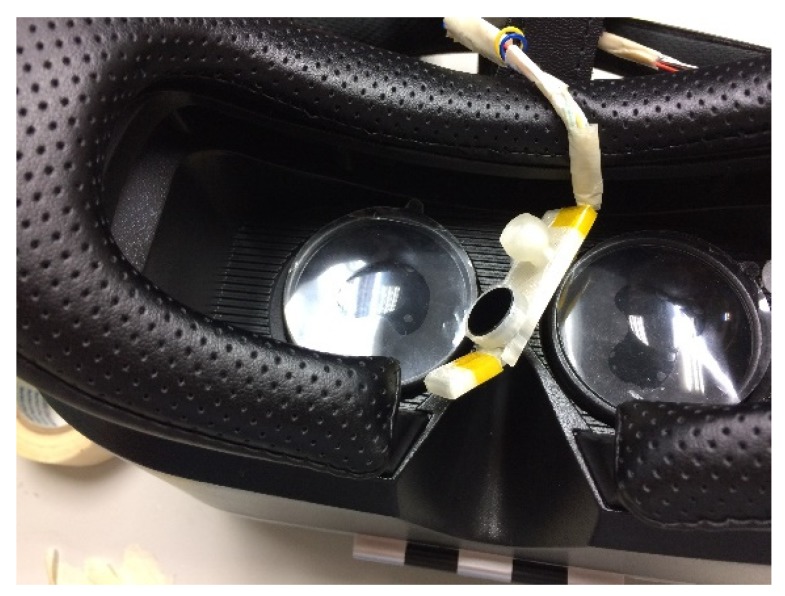
Comfortable space for the eye in the head-mounted device.

Several types of head-mounted devices are available for wearable eye trackers. It is necessary to place the camera close to the eyes to track eye movement (Figure 6). A device model with more space on the face was selected to avoid the eyes being too close to the light source and to increase the comfort. The camera module was placed under the eye to prevent the upper eyelid from obscuring the photographic line of sight. The placement angle affects the ratio of the image to the eye and the face and should be appropriate to capture a dark background.

The camera module was positioned at the center of the head-mounted device. This position resulted in less collision between the face and module and ensured a fixed angle. In addition, it is necessary to appropriately adjust the direction of the infrared light irradiation to avoid low brightness at the far side of the eye, which causes the pupil to be too close to the background color and results in difficulty in recognition.

**Figure 7:**
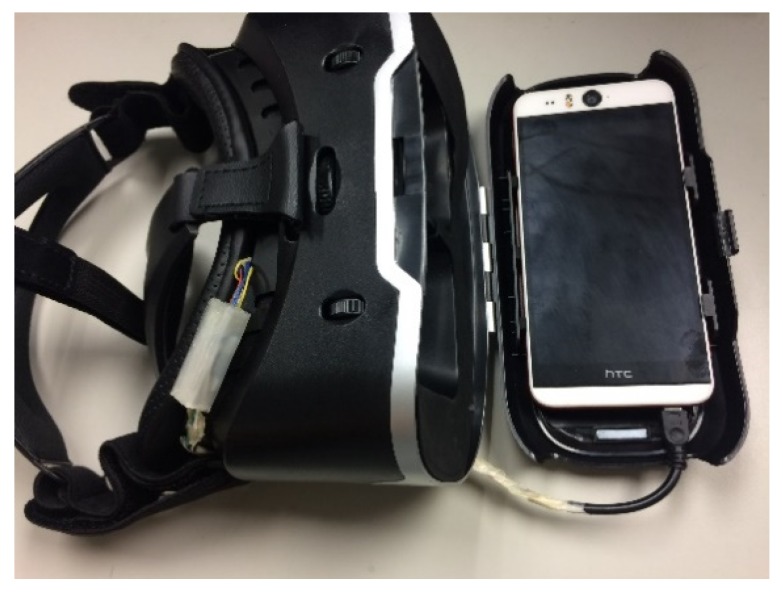
Mobile in the head-mounted device.

The camera module was attached to the center of the head-mounted device and finely adjusted according to the face of the user. As depicted in Figure 7, the phone was placed on the front phone slot and the camera cable was inserted into the micro USB connector of the phone. The phone slot was then closed. The user put on the device and adjusted the wearing position till he confirmed that he could see the blue safety range boundary of the screen.

The distance between the objective lens and the pupil distance was adjusted, and the field of view was approximately 90°–110°. The detailed specifications and description of the computer used in the study are shown in Table 1.

Please refer to Table 2 for detailed specifications of the mobile phone.

The hardware and electronic components of the proposed system are easy to obtain and relatively inexpensive, and the component names and costs (in US dollars) are listed in Table 3.

There are many consumer products on the infrared eye trackers, and the comparisons of these products and their prices are listed in Table 4.

## 4. Proposed Method

**Figure 8:**
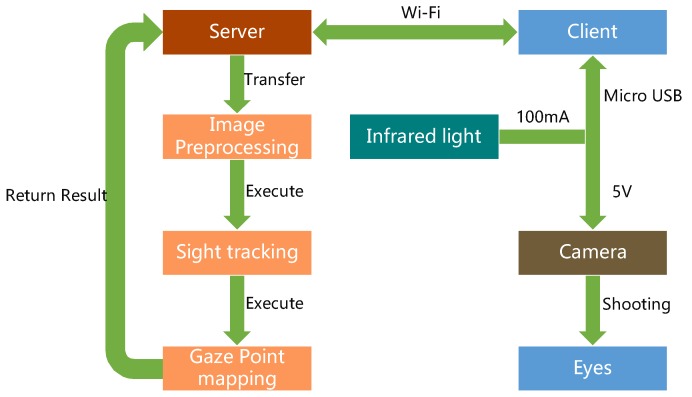
System architecture diagram [10].

The system architecture diagram is presented in Figure 8. First, in the client part, the camera captures the eye images that is exposed to infrared light and transmits it to the mobile phone through the micro USB interface. Then, the client program of the mobile phone transmits the image of the eye to the computer server using Wi-Fi, which is then passed to the processor. The processed image is returned to the server, and the server transmits the information to the mobile phone through Wi-Fi. In the server part, when the server obtains the images from the client, the preprocessing steps are then performed on the images to obtain clear ROI regions. Then the sights that form the pupils can be applied to build the eye model for mapping the gaze points onto the screen. The client program is developed using Java. The computer’s server is developed using Node.js. The image processing is developed using the C++ language and OpenCV library.

### 4.1. Preprocessing

**Figure 9:**
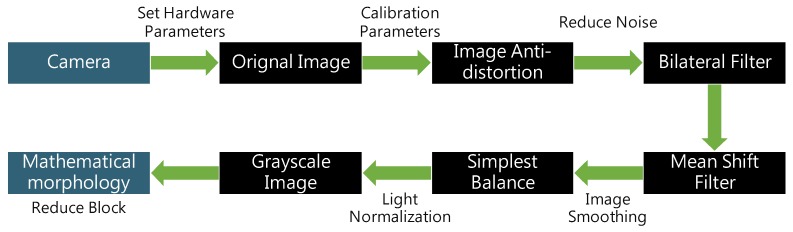
Image preprocessing flowchart.

The original image captured using a low-cost camera has substantial amounts of noise and low pixel stability. Therefore, multiple image preprocessing is required to render the image suitable for subsequent analysis. As depicted in Figure 9, preprocessing mainly involves three steps: 1. Setting of hardware parameters; 2. calibration of parameters; 3. image smoothing and light normalization.

#### 4.1.1. Setting of Hardware Parameters

**Figure 10:**
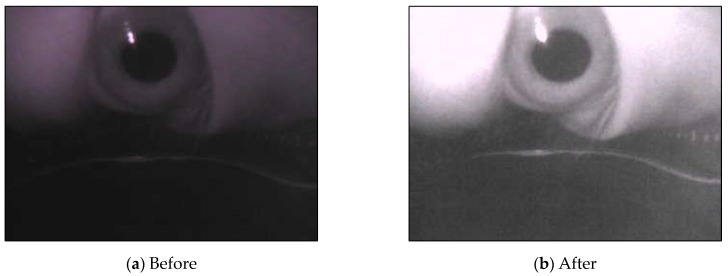
Results after setting USB video device class (UVC) parameters.

The image resolution transmitted by the camera was set to 320 × 240 pixels. The USB video device class (UVC) parameters were set to 50% brightness and 100% contrast, and the automatic white balance was set on. As depicted in Figure 10, after setting the hardware parameters, we can distinguish the background and the foreground in the original image. The boundary between the pupil and the iris was distinct, but these parameters reduced the eye reflection point.

#### 4.1.2. Distortion Calibration

**Figure 11:**
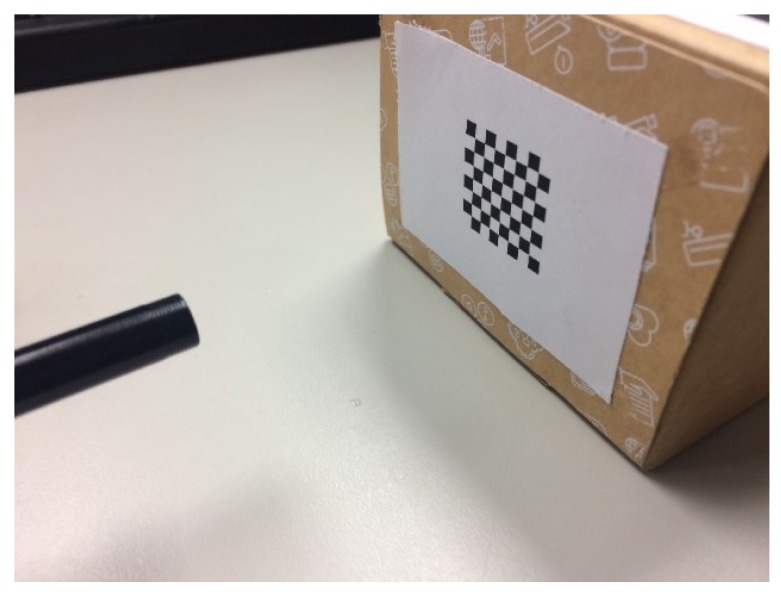
Use of the checkerboard image to correct the camera.

**Figure 12:**
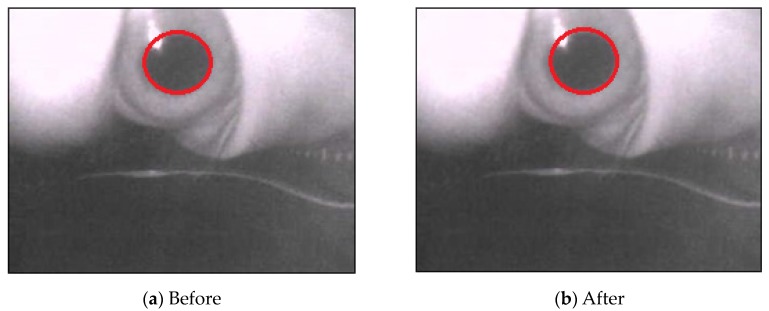
Results of distortion correction.

Each camera may have an image that may be deformed because of its hardware characteristics and production methods [22]. The camera was placed at a predefined distance to capture the image of the calibration object and the correction object was rotated to obtain the image of the correction object at various angles (Figure 11). The internal and extrinsic parameters of the camera can be calculated as follows:

The camera parameters obtained in advance were applied to the image for reverse deformation (Figure 12). Although the resultant image may be not so obvious for distortion correction, this step is important for accurately finding the pupil and correct ellipse regions, as depicted with the red circles in Figure 12.

#### 4.1.3. Image Smoothing and Light Normalization

Equation (1) and Equation (2) are used for the bilateral filter, where p is the target pixel and q is one pixel around the target pixel, Ip is the color of the target pixel, Iq is the color of a pixel around the target pixel, s is the pixel group around the target pixel, Gσs is the weighted pixels according to the distance, and Gσr is the weighted pixels according to pixel color difference.

(1) $$Ip=\frac{1}{Wp}\sum_{q\in s}G\sigma s(\left\|p-q\right\|)G\sigma r(\left|Ip-Iq\right|)Iq$$

(2) $$Wp=\sum_{q\in s}G\sigma s(\left\|p-q\right\|)G\sigma r(\left|Ip-Iq\right|)$$

**Figure 13:**
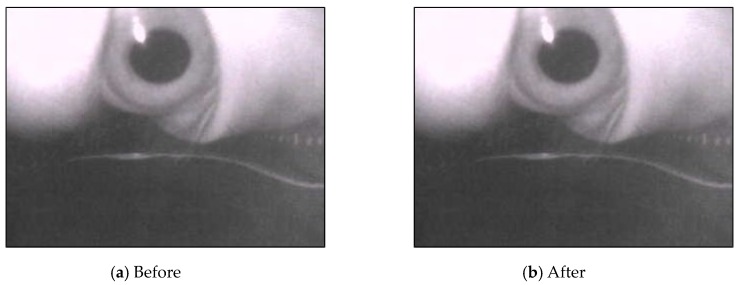
Results of bilateral filtering.

After calibrating parameters, we used bilateral filters to smoothen the image and preserve the edges of the image content that was suitable for retaining the pupil’s features (Figure 13).

**Figure 14:**
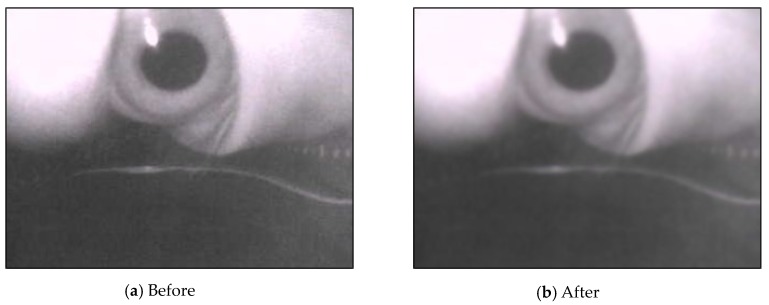
Results after using mean shift filtering.

Equation (3) is used for the mean shift filter. Here, $S_{h}$ is a high-dimensional sphere with a radius *h*. The image processed using the bilateral filter still had noise, with uneven coloring in the block. The mean shift filter was used to smoothen the pixels of similar colors in the image and preserve the edges of the image (Figure 14)

(3) $$M_{h}(x)=\frac{1}{k}\sum_{x_{i}\in S_{h}}\left(x_{i}-x\right)$$

**Figure 15:**
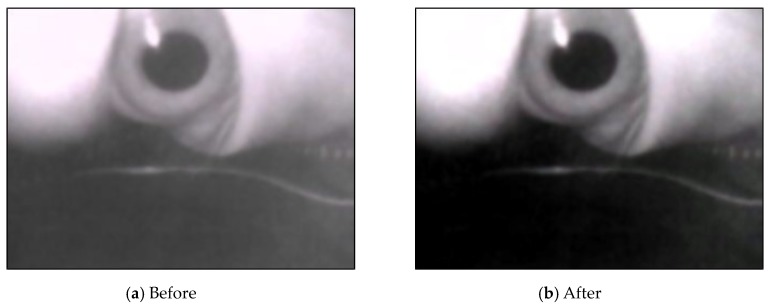
Results of the color balancing.

Because the eyes were close to the screen, the brightness of the screen affected the image light. The image threshold values obtained at various times were different from those of the binarization images. A simple color balance method [23] was used for light normalization. The processed image is depicted in Figure 15. The depth information of the pixel was used to convert the image into a grayscale image to reduce the number of calculations.

**Figure 16:**
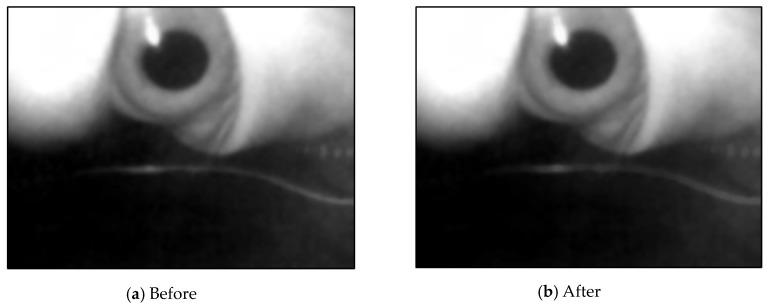
Image after applying mathematical morphological process.

Finally, mathematical operations were used to reduce the small noise in the image to ensure the contours in the captured image were as continuous as possible without fragmentation in subsequent analysis. Equation (4) and Equation (5) describe morphological erosion and dilation, respectively. Equation (4) describes the erosion operation, where f denotes grayscale images, b denotes structural elements, x and y denote coordinates, and s and t denote variables within the structural elements. Equation (5) describes the dilation operation, where b denotes the corresponding structural elements. The processed image is depicted in Figure 16.

(4) $$\left(f\Theta b\right)\left(x,y\right)=\underset{\left(s,t\right)\in b}{\min}\left\{f\left(x+s,y+t\right)\right\}$$

(5) $$\left(f⊕b\right)\left(x,y\right)=\underset{\left(s,t\right)\in b}{\max}\left\{f\left(x+s,y+t\right)\right\}$$

### 4.2. Capturing Pupil Image

The pupil should be isolated from the image post the processing of the image. This isolation can be divided into three steps, namely automatically removing the background, separating the front and the back scenes, and multithresholding to capture the pupil images.

#### 4.2.1. Automatically Removing the Background

**Figure 17:**
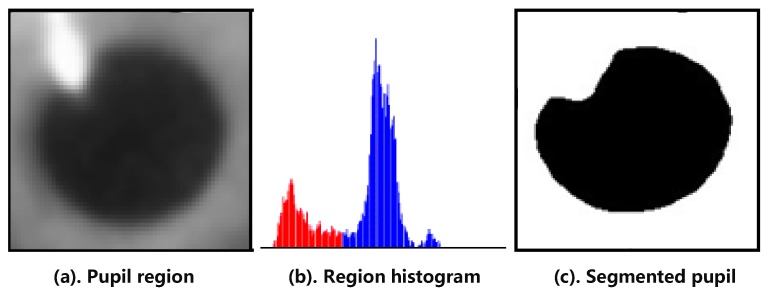
Binarization image representation using K-means [24,25].

In the preprocessed image, the lower half of the image was mostly the background. The pupil can produce an imperfect ellipse that may appear distorted in various directions for a particular angle rather than a simple circle. The black blocks in the image were searched and the largest black block was selected as the region of interest (ROI). Therefore, it is necessary to remove as much of the background as possible to avoid confusion in the background for a black block when capturing the pupil block. Next, a histogram of K-means [24,25] was used. The histogram was divided into two groups and binarization images using the central average of the two groups, as shown in Figure 17.

Next, the total number of pixels of different brightness in the image was determined to plot the image histogram [26] and redistribute the pixel values of the grayscale image.

**Figure 18:**
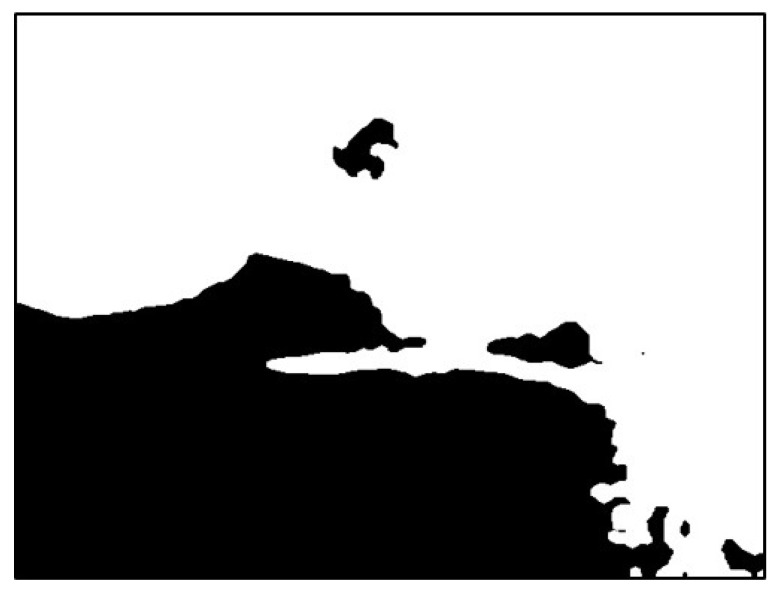
Result using dark group as the threshold.

**Figure 19:**
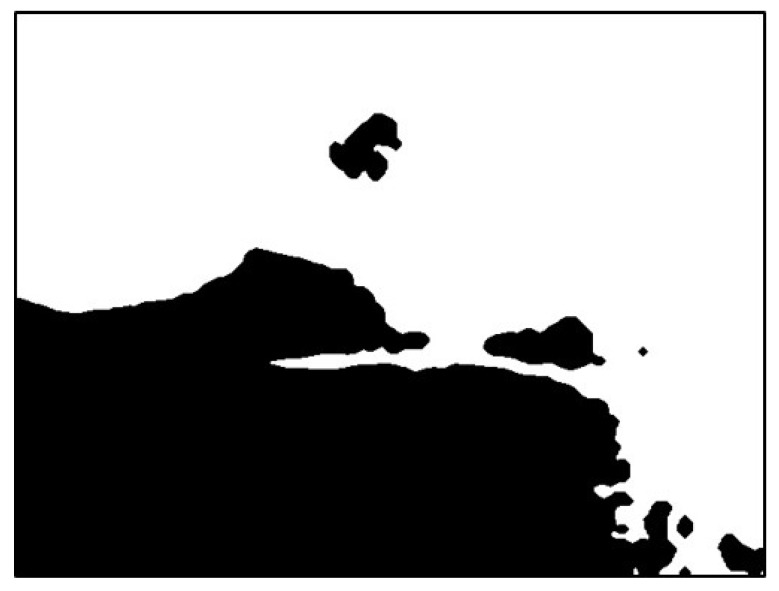
Result after erosion.

The image was extracted from the background value of the dark group as the threshold value binarization image, as depicted in Figure 18. To maximize the removal of dark values from the edges of the image, morphological erosion calculations were used. This was achieved by reducing white patches in binarization images, as illustrated in Figure 19.

**Figure 20:**
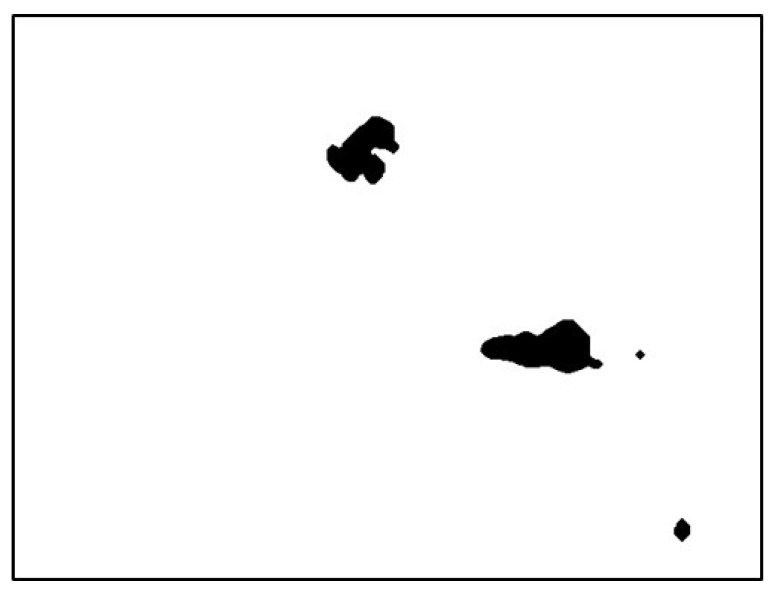
Result of filling the image with flood fill.

**Figure 21:**
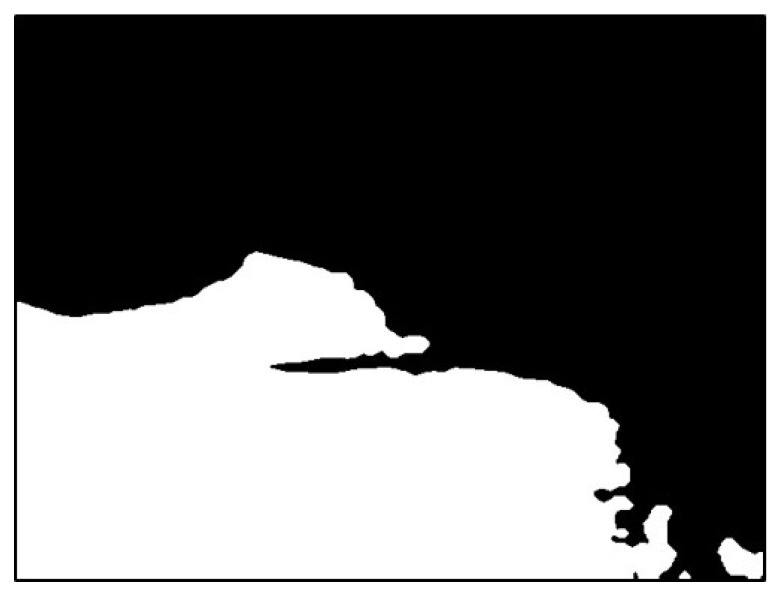
Result of subtracting the image before and after filling.

Next, the flood fill method was used to fill white pixels from the bottom row of black pixels in the image. Subsequently, this image was subtracted before filling to obtain the background mask (Figure 20 and Figure 21).

**Figure 22:**
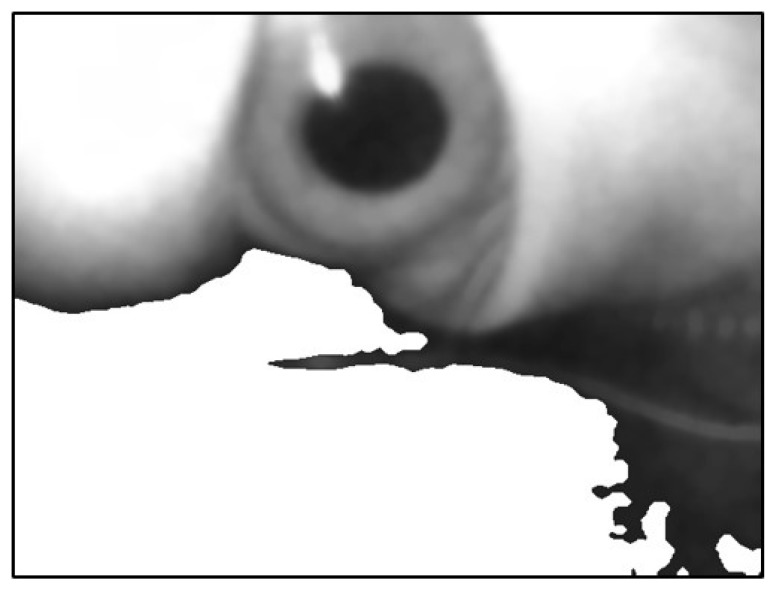
Superimposed mask image.

Finally, the mask image was overlain onto the original image to fill the background with white (Figure 22).

#### 4.2.2. Separating the Front and Back Scenes

**Figure 23:**
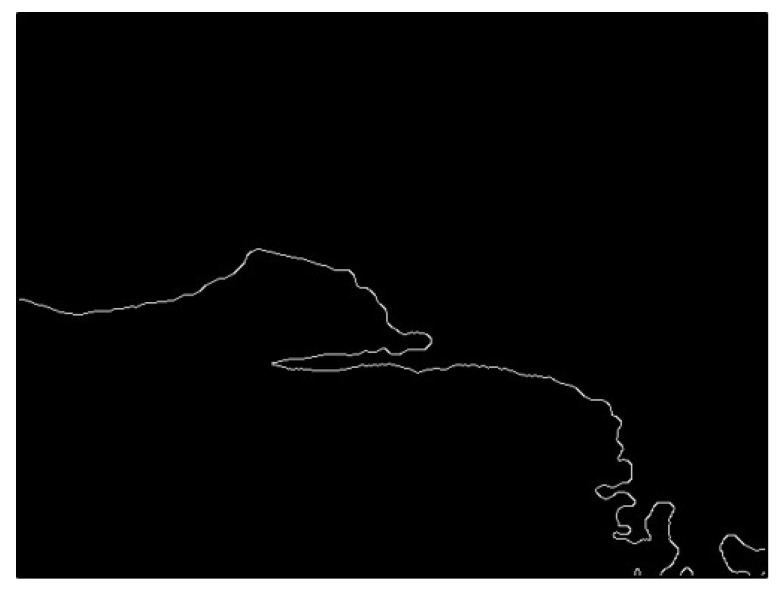
Image using canny edge detector.

The image that was automatically removed from the background still had some portions from the background. Therefore, the brighter blocks could not be distinguished using the threshold value. The canny edge detector was used to determine the edges of the image, as shown in Figure 23.

**Figure 24:**
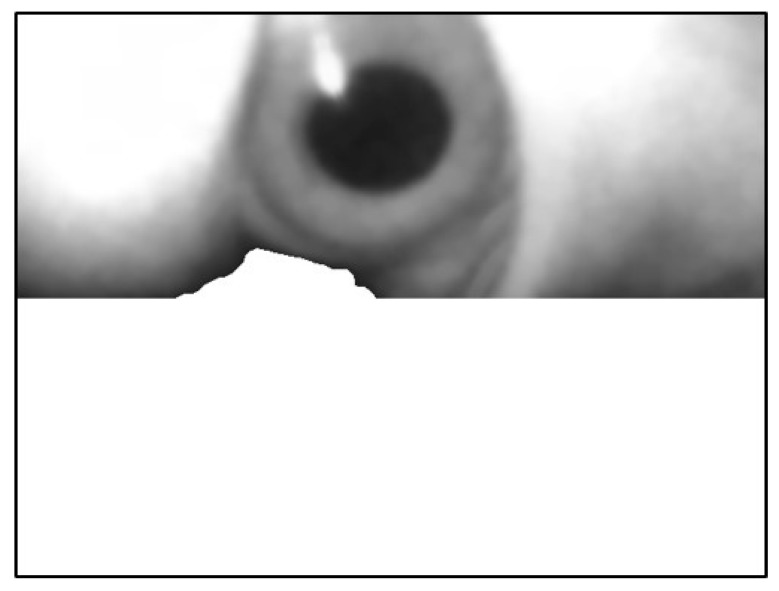
Filling the image in the lower part of the image.

The leftmost point of this edge contour was used as the starting coordinate of the background. Pixels ≥ this Y-value were filled with white, as depicted in Figure 24.

**Figure 25:**
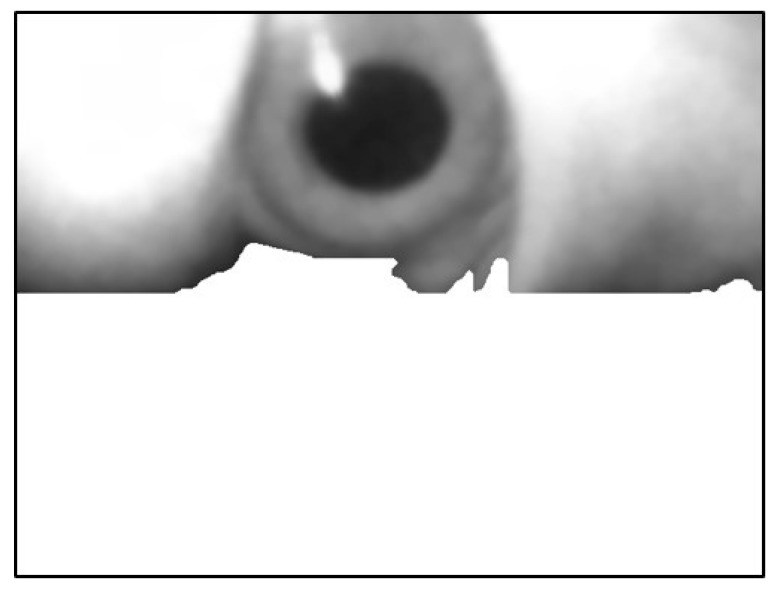
Automatically removed from the background the second time.

**Figure 26:**
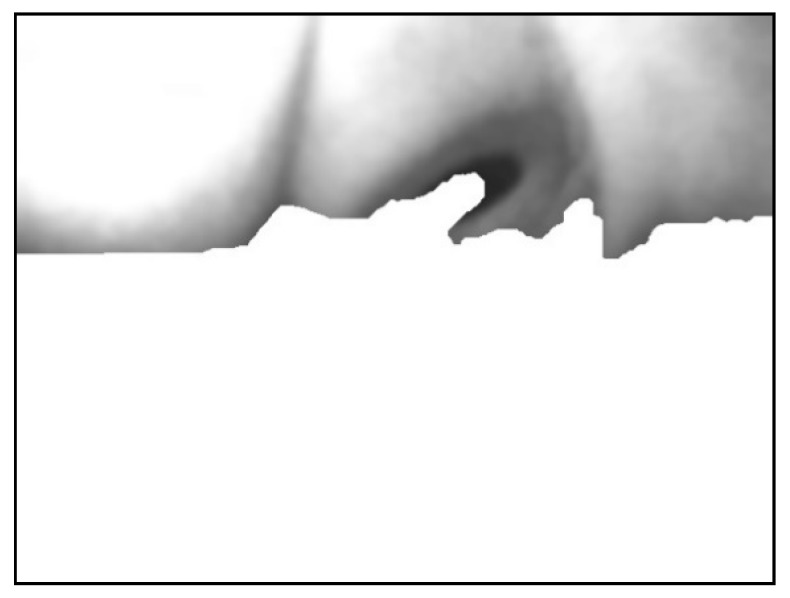
Image of connected pupil block.

The image still had dark pixels with residual background. These dark pixels can occupy dark blocks larger than what the pupil occupies and result in selection of incorrect ROI. To remove the background again, the flood fill method was used to fill black into the bottom row of white pixels from the image and then was automatically removed from the background, as depicted in Figure 25. These steps were repeated. However, the black block of the pupil can connect with the black region and result in removal, as shown in Figure 26. Therefore, a multiple automatic background removal stop method was proposed based on the maximum black block coordinates.

First, the largest dark block that was automatically removed previously from the background was identified and the width and height of the center coordinates were determined. Then, the center coordinates and width of the largest dark block in the image that was automatically removed from the background were determined again and compared with the Y-value of the previous center coordinates. If the Y-value of this dark block was higher than the Y-value of the previous dark block, the ROI was incorrect. Thus, the captured ROI should be closer to the image than the previous ROI. If the ROI moves down, then the automatic background removal should stop and the previous the image is the current foreground image and its largest dark block is the current ROI.

#### 4.2.3. Multithresholding to Capture the Pupil Images

The image was preprocessed and then automatically removed from the background. The front and back scenes were separated, and corresponding to the pixel value according to the cumulative amount in Equations (6) and (7) the H(j) of the pixel was used in the image, and the multithresholding [27,28,29] was considered to isolate the pupil portion.

(6) $$H^{\prime }\left(i\right)=\underset{0\leq j<i}{\sum }H\left(j\right)$$

(7) $$\mathrm{equalized}\left(x,y\right)=H^{\prime }\left(src\left(x,y\right)\right)$$

**Figure 27:**
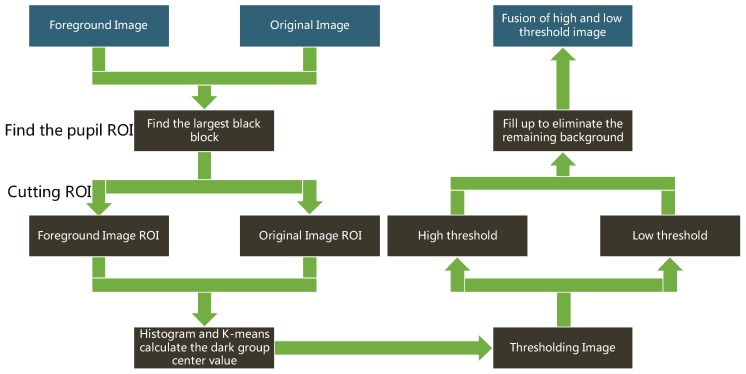
Flowchart of capturing pupil images [10].

**Figure 28:**
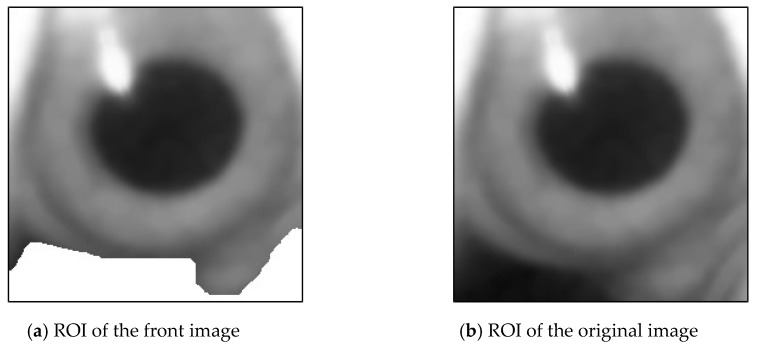
Region of interest (ROI) of the image.

Figure 27 shows the flowchart of capturing pupil image [10]. The pupil image may contain brighter reflective points and darker pixels away from the light source. Therefore, a single threshold cannot be appropriately used to identify a pupil. The high and low thresholds were used as intervals to combine the results of binarization of the two thresholds of the pupil image which use the H(j) in Equations (6) and (7). The ROI of the foreground image was used to obtain the dark group center value as the high threshold (Figure 28a). The image of the unremoved background was used to obtain the dark group center value as the low threshold, as shown in Figure 28b.

**Figure 29:**
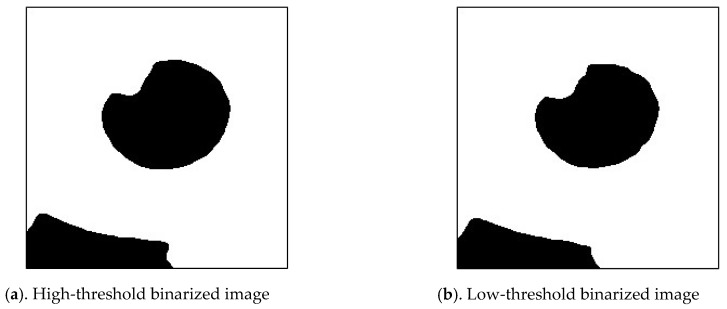
Image Binarization.

A binary image of the pupil was obtained using the high-threshold value. Similarly, a binarization image of the pupil was obtained from the original image using the low-threshold value, as depicted in Figure 29a,b.

**Figure 30:**
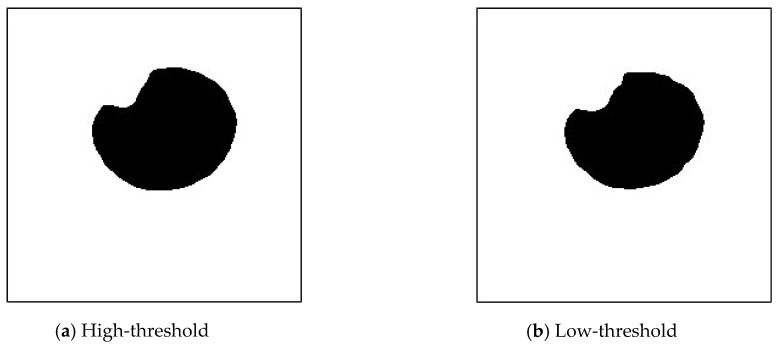
Image using the flood-filling method.

Next, the flood-fill region labeling method was used to transform the black pixels from the bottom row of the image into white. The results are depicted in Figure 30a,b.

**Figure 31:**
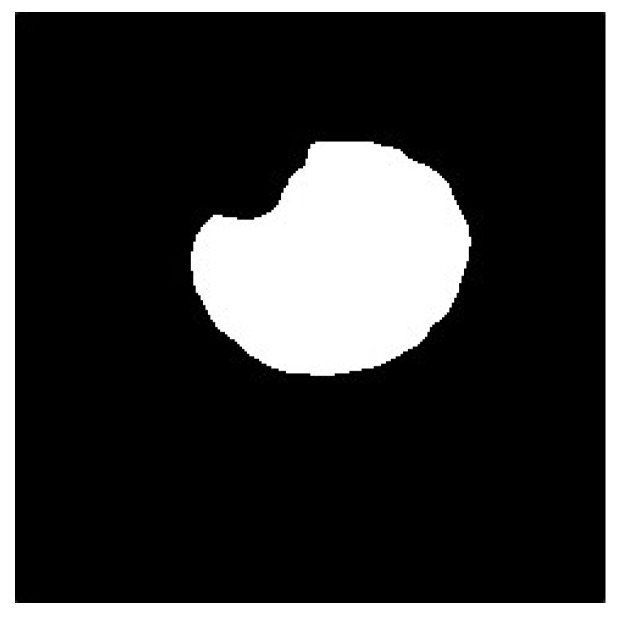
Anti-whited low-threshold image.

**Figure 32:**
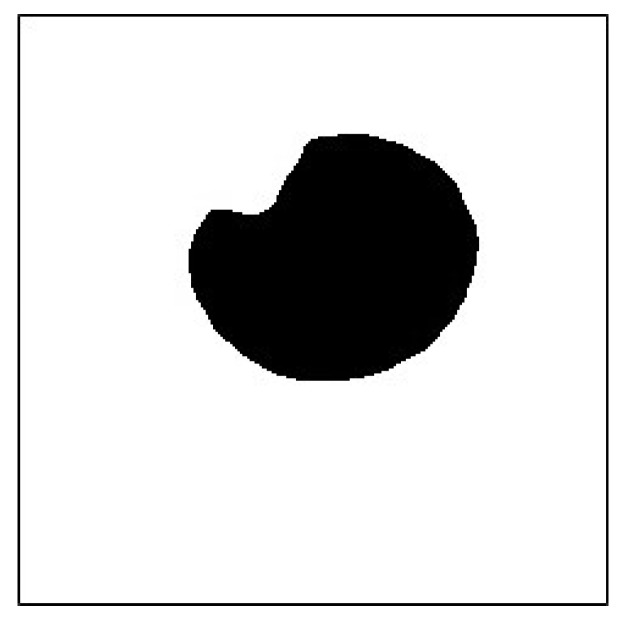
Multithreshold pupil image.

Finally, to preserve the black pixels in the image as much as possible, it is necessary to fuse the pupil high- and low-threshold images. After subtracting the low-threshold image and subsequently subtracting the low-threshold image from the high-threshold image, the resultant multilevel thresholds were used to capture the pupil image, as depicted in Figure 31 and Figure 32.

### 4.3. Gaze Point Mapping

**Figure 33:**
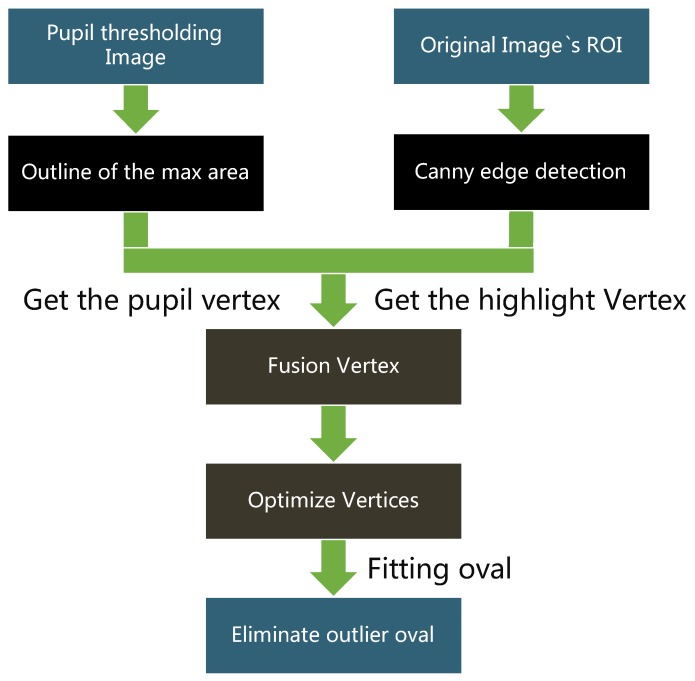
Flowchart for fitting the pupil ellipse [10].

Figure 33 shows the flowchart for fitting the pupil ellipse [10]. The ellipse closest to the shape of the pupil was fit using multiple pupil and ROI images.

#### 4.3.1. Combining Pupil Contour and Reflective Highlight Contour

**Figure 34:**
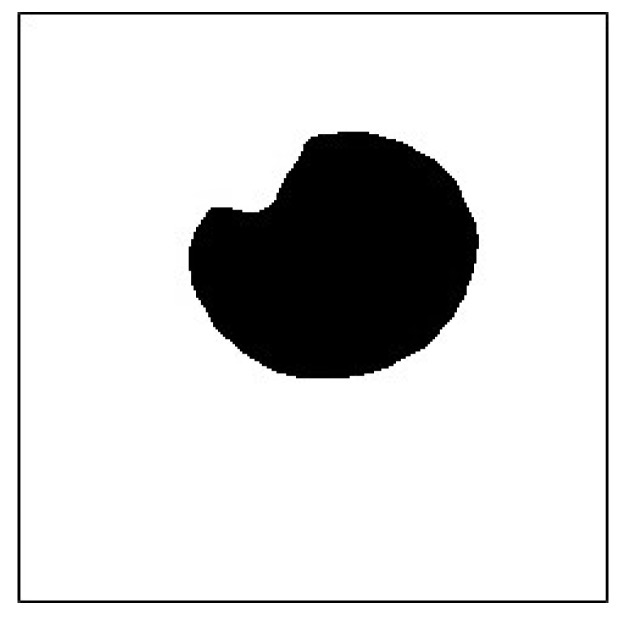
Pupil image use multithresholds.

**Figure 35:**
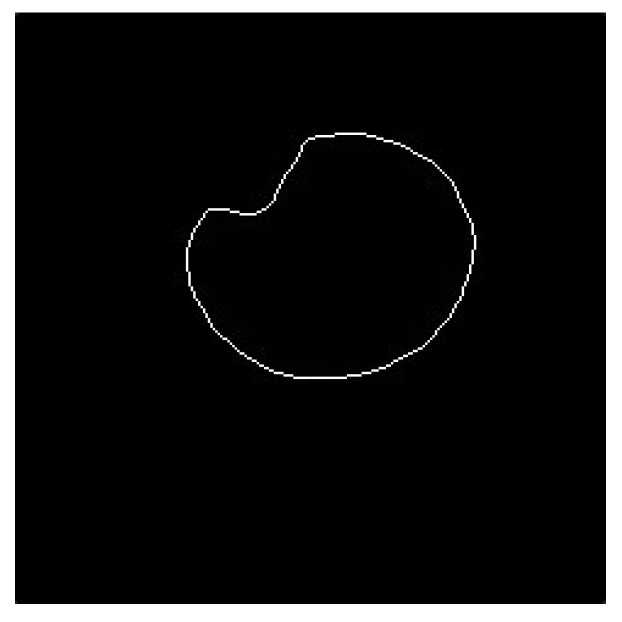
Maximum area contour.

Topological structural analysis of digitized binarization images by using border contours was performed according to [30] to determine the contours in the pupil image captured using multithresholding. Then, the area of all the contours was determined and the contour with the largest area was identified. The vertex of the largest area contour was considered as the pupil silhouette vertex (Figure 34 and Figure 35).

**Figure 36:**
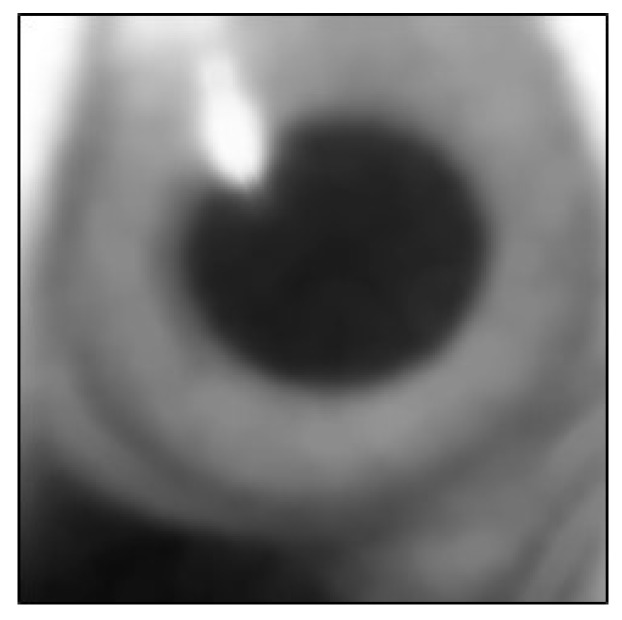
Original image.

**Figure 37:**
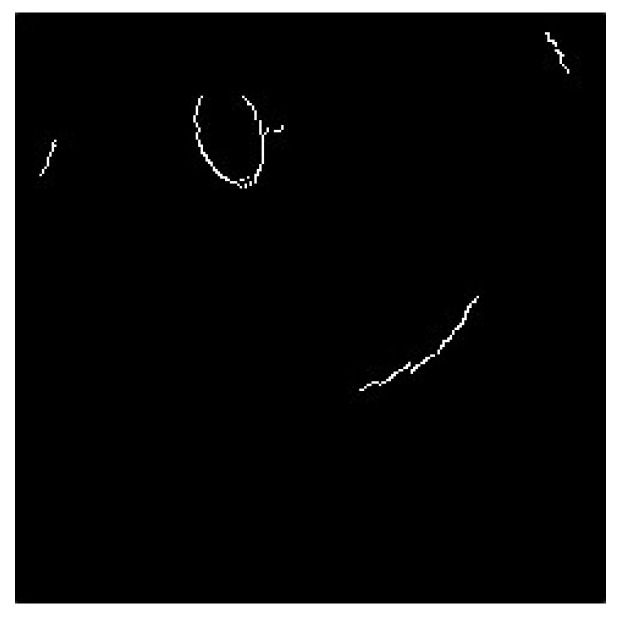
Reflective highlight contour.

The pupil contains reflective highlights that affect the accuracy of the fitted ellipse. Therefore, the highlight vertices of the highlights were added to the vertices of the pupil contour. First, the canny edge detection method was used on the original image ROI to determine the edge contour in the image. The vertices of these edge contours were used as the vertices of the reflective highlights. This is depicted in Figure 36 and Figure 37.

**Figure 38:**
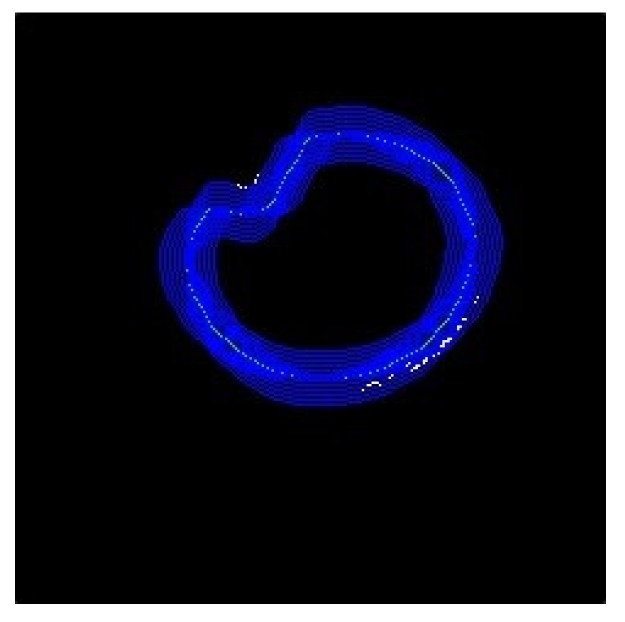
Coincident contour vertex image.

Next, the new coordinates of the vertices of the reflective highlight image and the X-value and the Y-value were increased and decreased several times. The recombination of the new coordinates with the highlights of the reflective highlights was verified. The vertices obtained using this method were added to the pupil vertices (Figure 38).

#### 4.3.2. Optimizing Pupil Vertices

**Figure 39:**
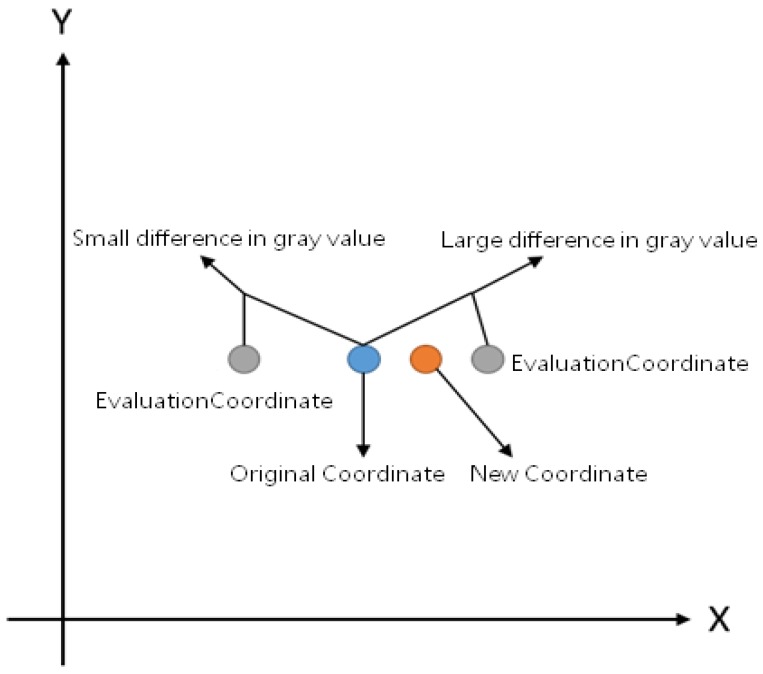
Schematic of optimized pupil vertices.

The optimization steps of the pupil vertices are depicted in the Figure 39. First, all the vertices of the pupil contour were sequentially removed, and the coordinates of the pixels were used to obtain the gray value of the pixel on the grayscale image (the blue circle). Then, its coordinate X-values were increased, and two new coordinates were used to obtain the gray value of the pixel on the grayscale image (the gray circles). This gray value of the original coordinate was compared with that of the original to obtain the absolute difference value. This difference value was multiplied by its coordinate X-value, and after performing this operation multiple times, the average of these values was used to calculate the coordinate X-value. This method was also applied to the coordinate Y-value. These coordinate X- and Y-values become the new contour vertex coordinates (the orange circle), which yield an average coordinate based on the grayscale difference values around the pixel.

#### 4.3.3. Fitting Ellipse and Eliminating Outliers

**Figure 40:**
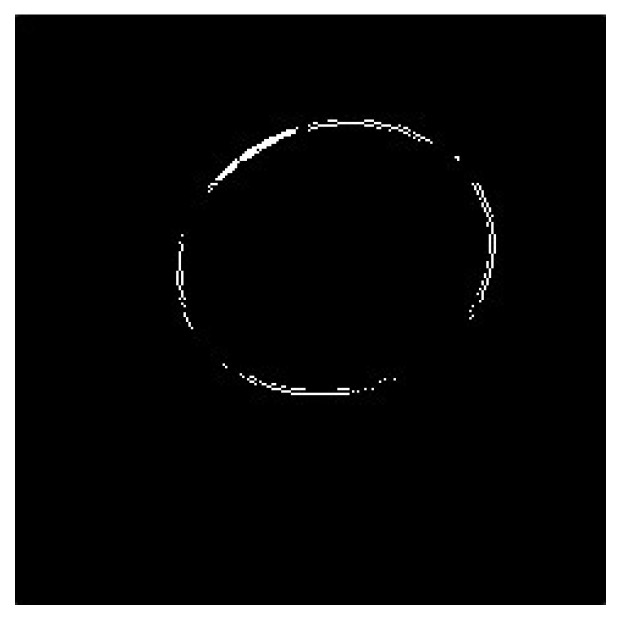
Pupil elliptical vertex image.

**Figure 41:**
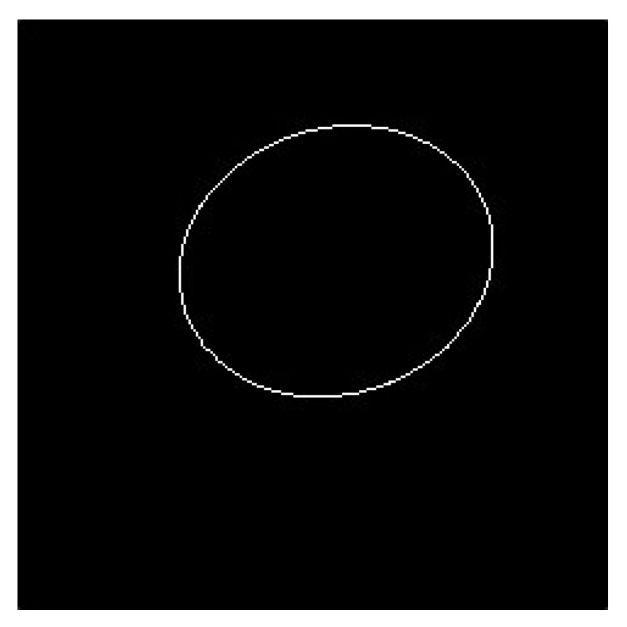
Fitting pupil elliptical image.

First, an ellipse was fitted using the current pupil silhouette vertex. If the center coordinates of this ellipse were not within the current pupil ROI, then the ellipse was removed. If the ellipse center coordinates fit the ellipse within the current ROI and the ellipse center coordinates were within the pupil contour, the ellipse was used as the pupil ellipse (Figure 40 and Figure 41).

**Figure 42:**
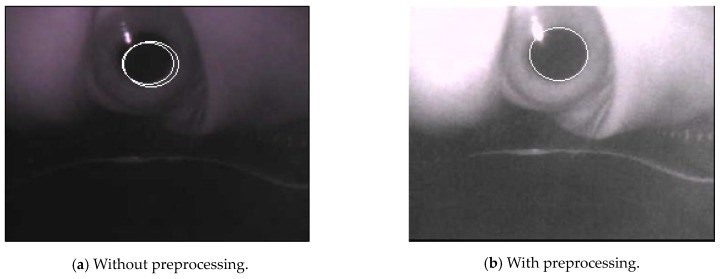
The pupil elliptical detection results with the proposed preprocessing techniques.

As mentioned above, the preprocessing is to accurately locate the pupil regions to obtain satisfactory gaze tracking and point estimation results. As shown in Figure 42, we can compare the resultant pupil elliptical regions with and without the proposed preprocessing methods, and we can see that without the preprocessing, the pupil elliptical regions cannot be appropriately located. In Figure 42a, the detected pupil elliptical region without preprocessing reveal that the two confused pupils are located and they are not in the correct positions. Therefore, the proposed preprocessing method can efficiently promote the results for finding the pupil regions for gaze tracking and point estimation.

### 4.4. Establishing Eye Model

To obtain the line-of-sight vector corresponding to the pupil ellipse, the eyeball model should be established first. The sight tracking method based on the shape was used in the study [9].

**Figure 43:**
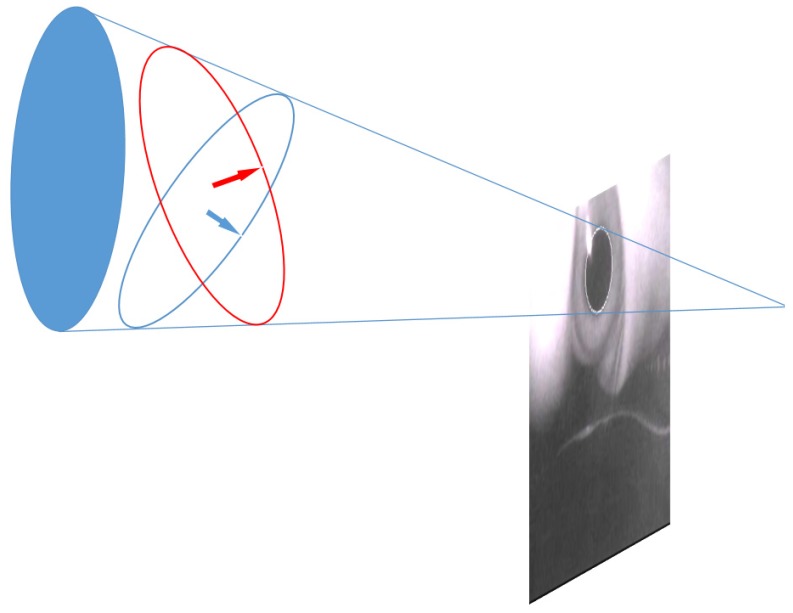
Pupil elliptical reflection projection [9].

First, each pupil’s ellipsoid reflection was projected into a circle. As depicted in Figure 43, a cone was created through the focus of the camera and the pupil ellipse on the image plane. Two possible circles could be solved in the cone, and the normal vector of the circle was the hypothetical gaze point vector.

**Figure 44:**
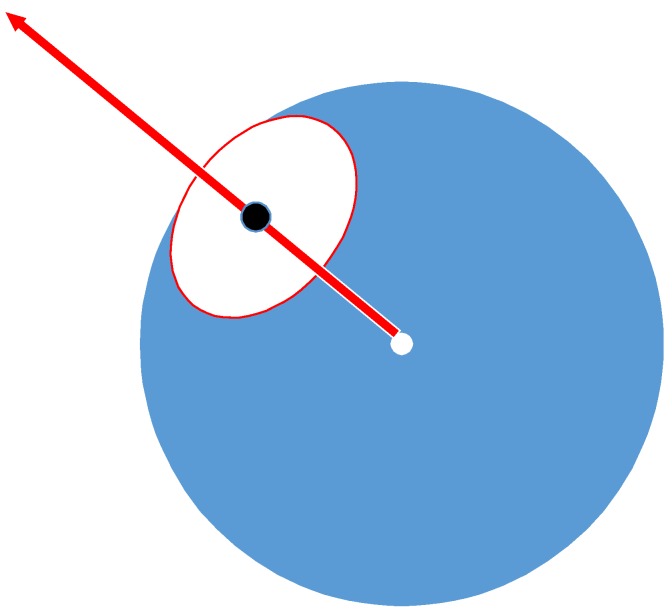
Eyeball model.

Assuming that the sphere was the same as the center of rotation of the eyeball, the line of sight was the normal vector of the disk and the radial vector extended from the center of the sphere to the center of the pupil (as depicted in Figure 44).

The intersection of all projected gaze point vectors was considered the center of the sphere. The center of the projected sphere depends on the intersection of these lines of sight.

**Figure 45:**
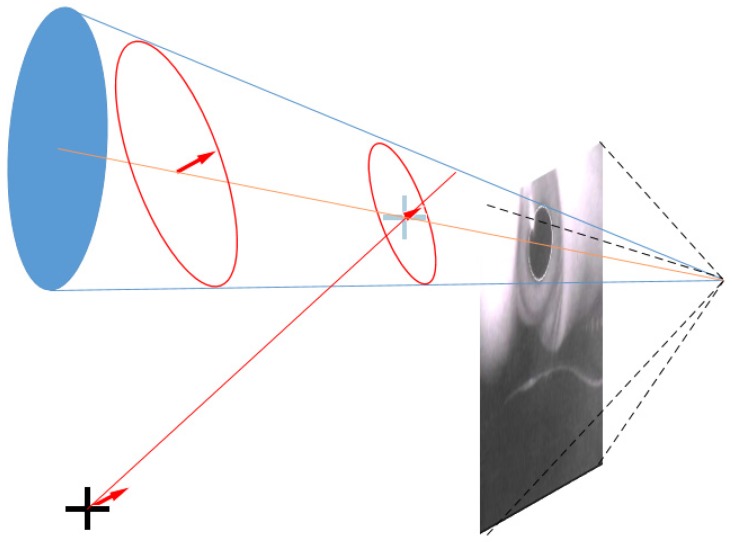
Center of the sphere intersects the gaze point vector [9].

The center of the projected sphere was thus obtained (Figure 45) for each pupil circle (red circle), the possible back projection center (orange line) was considered and a line to the line of sight from the center of the sphere was obtained. The intersection of the blue line with the line from the center of the sphere produced an orange fork, which was the center of the circle (red dotted circle) on the tangent to the sphere. The radius of the sphere was the distance from center of the sphere (blue fork) to the center of the circle.

### 4.5. Establishing the Mapping Model

First, the boresight ellipse is the reflection projected to the line of sight vector, and the vector is mapped onto the screen plane to obtain the gaze point coordinates. The correspondence can be expressed using the following equations:

(8) $$S_{x}=a_{0}+a_{1}x+a_{2}y+a_{3}z+a_{4}xy+a_{5}yz+a_{6}xz+a_{7}xyz+a_{8}x^{2}+a_{9}y^{2}+a_{10}z^{2}\;S_{y}=b_{0}+b_{1}x+b_{2}y+b_{3}z+b_{4}xy+b_{5}yz+b_{6}xz+b_{7}xyz+b_{8}x^{2}+b_{9}y^{2}+b_{10}z^{2}$$

**Figure 46:**
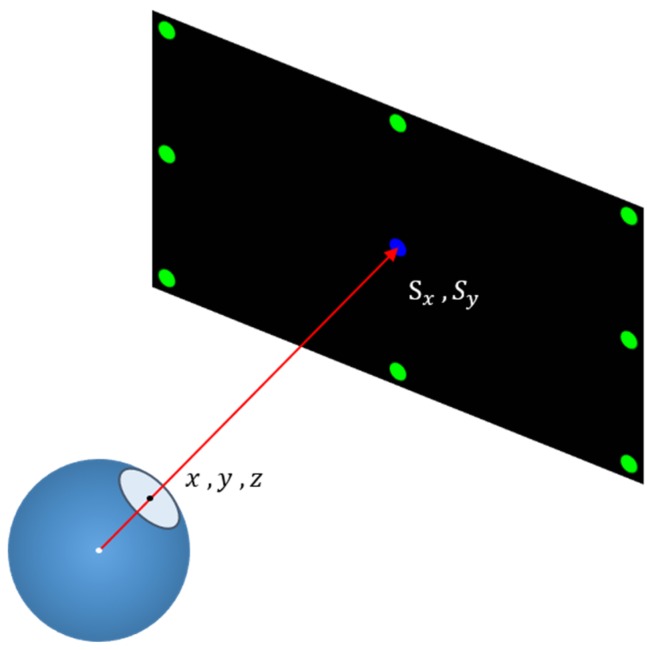
Line of sight vector versus screen coordinates.

In Equation (8), $S_{x}$ and $S_{y}$ are the coordinate points on the screen and *x*, *y*, and *z* are the sight vectors captured by the camera. Each equation has 11 constants. Therefore, at least 11 equations are required to solve the constant group, and they represent that the relationship between the line of sight vector and the screen coordinates should be corrected through the calibration process (Figure 46). In this thesis, the singular value decomposition [31] was used to solve the aforementioned equation constant group.

## 5. Experiment Results

### 5.1. Experiment Device and Environment

The performance of eye detection, slight detection, and fixation error calculation was evaluated. In these experiments, gaze tracking and point estimation of low-cost head-mounted devices were tested for four users.

Precision, recall, and accuracy were calculated. In addition, experiments on eye, sight, and fixation error calculation were conducted.

This study was conducted in the same environment for all four subjects, and the same scene was repeated every time. The subjects aged from 22~40 and the gender are all males, they have no eye diseases but myopia with 2.0 diopters. Open eye detection rate and eye-tracking accuracy were tested. The detection rate had four calculation parameters: true positive (TP), which indicates that both the actual situation and detection are positive samples; true negative (TN), which indicates that both the actual situation and detection are negative samples; false positive (FP), which indicates that the actual situation is a negative sample but the detection is a positive sample; and false negative (*FN*), which indicates that the actual situation is a positive sample but is detected as a negative sample. If a positive sample occurs, then the event has a value of one and the negative sample is allotted a value of zero. If a positive sample is detected, then the detection is denoted as one and the negative sample as zero.

(9) $$\mathrm{Accuracy}=\frac{TP+TN}{TP+TN+FP+FN}$$

(10) $$\mathrm{Precision}=\frac{TP}{TP+FP}$$

(11) $$\mathrm{Recall}=\frac{TP}{TP+FN}$$

In Equation (9), accuracy is the ratio of the correct detection of all samples. In Equation (10), precision is the ratio of positive samples of events in all positive samples detected. In Equation (11), recall is the ratio of the positive samples detected in the positive samples of all events.

### 5.2. Experimental Method

**Figure 47:**
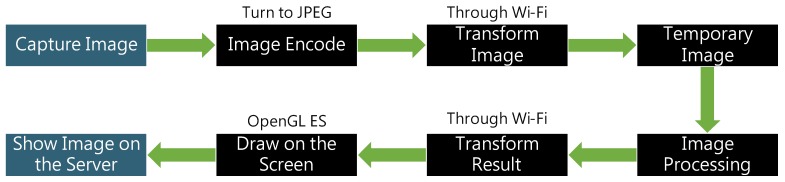
Flowchart of the system.

The processing steps of the proposed method is shown in Figure 47. First, the computational timings of the proposed head-mounted device and processing methods are given as the following analytical results. The mobile phone encoded the captured image, and the average processing time of each image was 2 ms. The image was transmitted from the mobile phone to the computer and transmission of each image was completed in an average of 50 ms. The computer temporarily stored the image to the disk for an average of 2 ms. Image processing was performed on the computer. The average processing time per image was 0.018 ms. The processed images are transferred from the computer to the mobile phone. This transmission back to the mobile was completed in an average of 50 ms. The phone presents the results on the screen, with every image presented in an average of 5 ms. The total average processing time of the system is about 110 ms.

The experimental method was divided into calibration and verification given as the following subsections.

#### 5.2.1. Calibration Process

The calibration process is divided into a correction eyeball model and a correction mapping model. A pupil image is required for the correction eyeball model and the correction mapping model requires a line of sight vector and screen coordinates. This calibration process was required to achieve short calibration times and enhance user experiences. This study combined the two calibration models including an eyeball correction model and a mapping correction model.

**Figure 48:**
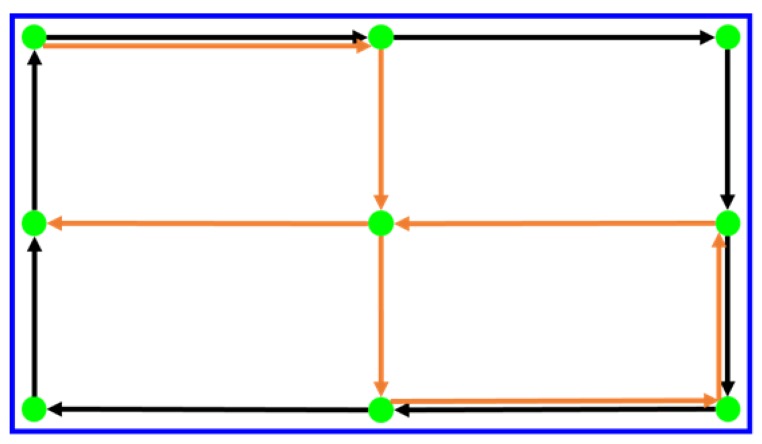
Integrated calibration diagram.

As depicted in Figure 48, this integrated calibration process relies on the user viewing the green dot on the screen and following its movement. The first time is to perform the eyeball correction model. From the top left corner, the eyeball model is developed according to the direction of the black arrow and back to the top left corner finally. Then the mapping correction model is calibrated based on the direction of the orange arrow. The blue range at the edge of the screen indicates the maximum range that the green dot can reach. When the user wears the head-mounted device, it is necessary to confirm that the blue frame can be clearly seen.

#### 5.2.2. Verification Process and Method

**Figure 49:**
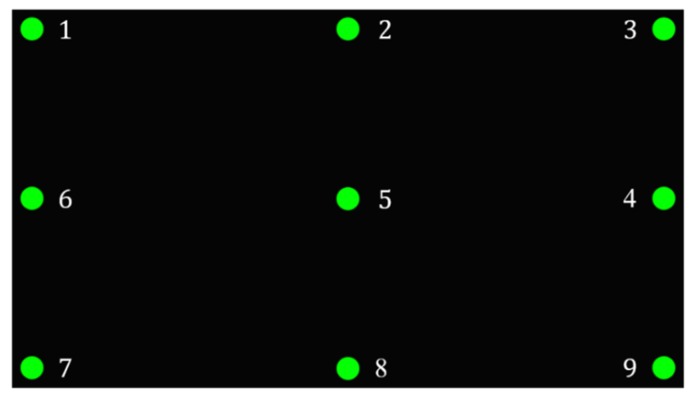
Gaze point sequence.

As shown in Figure 49, after the calibration process, we verified the line of sight tracking and determined the gaze point. The user gazed at each green point in sequence for 6 s. In the first 3 s, the eyes were allowed to move and stabilize. The image and coordinates were captured and returned for sampling.

The pixel density of the screen differs between various experimental devices and environments. The pixel distance cannot be used as the error unit. The distance between the user and the screen is also different. The unit distance cannot be used as the unit of error. Therefore, it is necessary to convert the error distance into an error angle to obtain a standard error unit.

**Figure 50:**
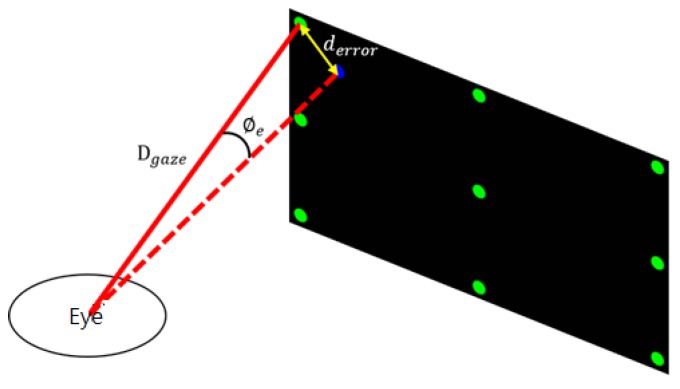
Error angle diagram.

As depicted in Figure 50, $d_{error}$ is the distance between the ideal gaze point and the actual gaze point and $D_{gaze}$ is the distance between the subject and the ideal gaze point. The error angle $\emptyset_{e}$ can be obtained as follows:

(12) $$\emptyset_{e}^{}=\tan^{-1}\left[\frac{d_{error}}{D_{gaze}}\right]$$

### 5.3. Performance Evaluation of Eye Detection

**Figure 51:**
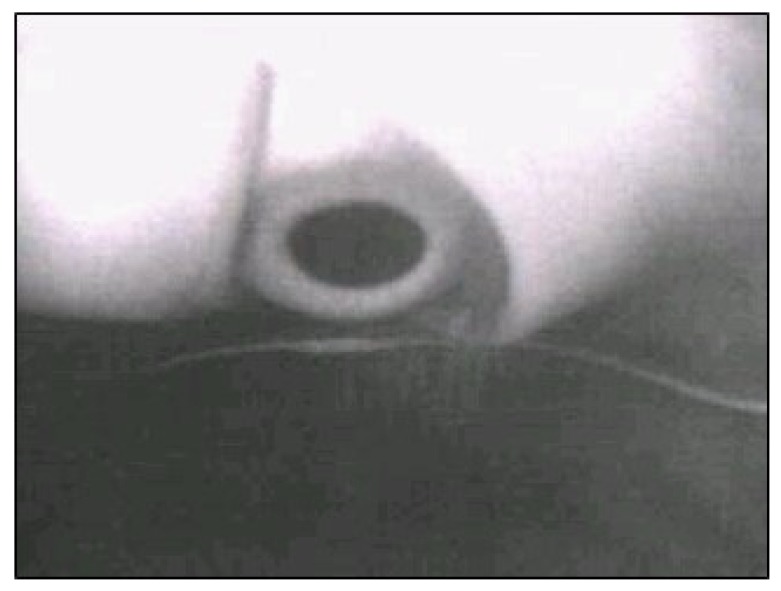
Infrared light image of the eye.

This study designed the experiments on eye detection based on Dong et al. [32] study. This study adopted the head-mounted devices, while the studies of Jen et al. [16], Dobeš et al. [17], and Lee et al. [18] used the desktop environments for eye tracking. The experiments are compared to those studies conducted by Lee et al. [18], Jen et al. [16], and Dobeš et al. [17]. Figure 51 presents the infrared light image of the eyes. Users looked at nine points on the screen and moved their heads accordingly. This experiment records the eye videos and uses each frame of the videos as the samples. The frame rate of these videos are 30 frame per second (FPS).

Table 5 lists the number of sample for four users. Sample number of nine gaze points on the screen for the user. The results show that there are almost the same number of samples at these nine points.

This eye detection experiment compares the eye detection accuracy, precision, and recall with the study of Lee [18], Jen [16], and Dobeš [17]. Table 6 lists the results of open eye detection rate for four users, which reveal that the average accuracy, precision, and recall rates can exceed 97%. The results of accuracy shows that the average are better than Jen [16] and Dobeš [17] and less than Lee [18]. The results of precision and recall rates shows that the average are better than others.

**Figure 52:**
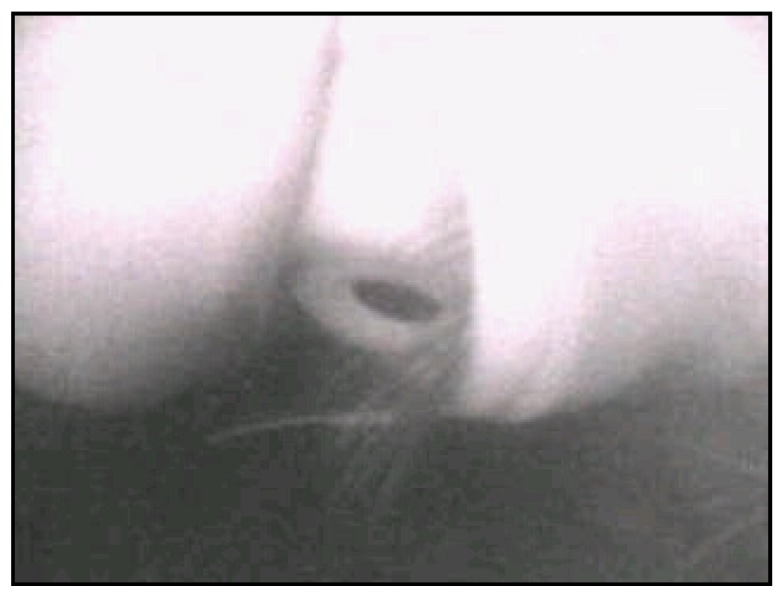
Eyelashes block the pupil.

**Figure 53:**
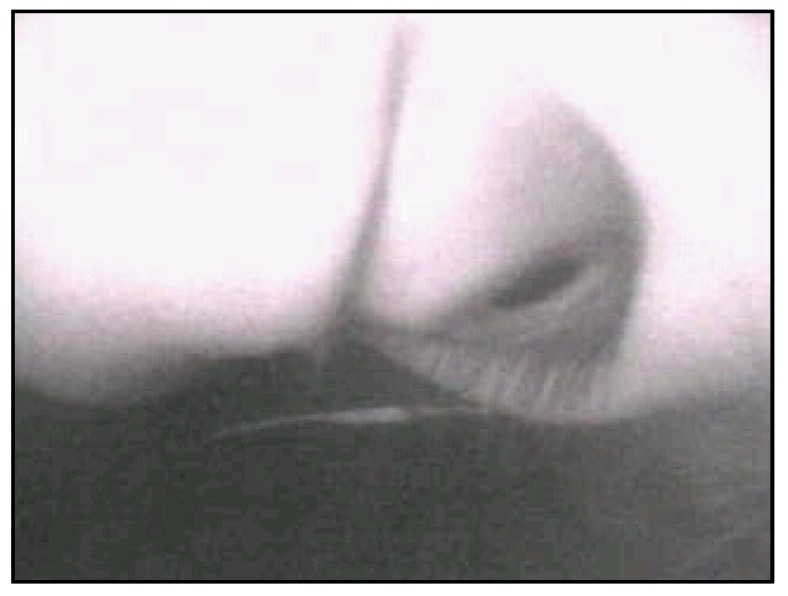
Too many dark pixels in the eye socket.

Because the eyelashes of User2 blocked the pupil, the pupil was removed in the binarization image in Figure 52. Figure 53 depicts the case wherein the eye-socket dark-pixel area of the user was larger than the pupil, resulting in the selection of the wrong ROI. Therefore, we can find that the sizes of eyelashes are no more than one centimeter, and so that the detection accuracy (User 2) is not significantly influenced and still provide a satisfactory accuracy rate.

### 5.4. Performance Evaluation of Slight Detection

Table 7 lists the results of sight detection rate for four users, which reveal that the average accuracy, precision, and recall rates can exceed 89%.

**Figure 54:**
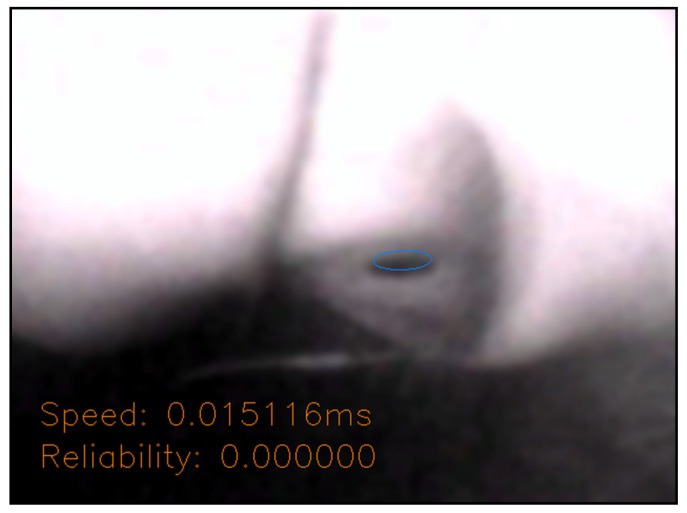
Dark pixels of the pupil are too less or unstable shape of ellipse.

As depicted in Figure 54, when the eyes were far away from the angle of view of the camera, fewer dark pixels of the pupil were captured or the shape of the pupil ellipse was unstable, resulting in insufficient credibility.

### 5.5. Performance Evaluation of Fixation Error

Table 8 lists the results of fixation errors for the users. The overall fixation point error had an accuracy of less than 3.2° and precision less than 1.2°. However, Point 1 is less accurate than other gaze points because the position of the gaze point and the eye tracking for the subject were not fully confirmed. Adjusting the calibration process can overcome this error. In addition, measurements at Point 5 proved more accurate than other gaze points because Point 5 was least susceptible to the field-of-view errors of the headset in the center of the screen. Point 5 is also less susceptible to the error of the lens of the head-mounted device. Therefore, the subject could gaze at this point easily and intently.

### 5.6. Satisfaction Surveys of Users

Table 9 lists the results of surveys for the users. After the experiment, this study invites users to conduct satisfaction surveys and interviews on the satisfaction of head-mounted devices. It consisted of six items with a five-point rating scheme (from 1-strongly disagree to 5-strongly agree), such as “ease of use,” “easy to navigate,” “adaptively useful,” “sufficiency,” “enjoyment,” “useful.” The average of the user satisfactions are higher than 4.25. In the part of the interview, some of their suggestions include: “The device should be bigger. Although the device allows no glasses, but if I want wear the glasses, the device is too tight,” “I have adjustment every time when I wear the device,” “I feel so hot when I put on the device,” “It is too heavy and it hurts my nose.” In the future, suggestions will let us to improve the implementation of the device.

## 6. Conclusions

The proposed eye-tracker system adopts the infrared light as an illumination source. Therefore, a dark head-mounted device can be used. The sight line detection of the proposed method does not rely on the reflections on the eyeball, pupil, or the coordinates of the center of the iris in the eye frame. The eyeball model is established using the proposed calibration process. Therefore, the shooting angle is not limited as long as the eyes can be completely captured. The evaluation of the gaze point is calculated using the visual line vector, and the mapping model is established using the calibration process and not limited by the distance between the eye and the screen. The average eye and sight detection rate are both over 89%. With regard to the gaze point error, the average gaze points’ accuracy is less than 3.2°, and the average gaze points’ precision is less than 1.2°.

The screen of the head-mounted is fixed at a distance to the eyes and is not affected by the rotation and movement of the head. Therefore, the use of the chin support frame can be eliminated, which is more flexible and convenient. The eye tracker in the headset only requires a camera module, a mobile phone, and a computer to track the line of sight and map the gaze point. These hardware components are cost-effective and easy to obtain, thereby reducing the overall cost of the system. The system cost is lower than the products available in the market.

Although it can be found from the experimental results that the eyelashes may affect the accuracy rate. Gabor filter [33] will be added in the future to grab and remove the eyelashes to increase the accuracy rate. This accuracy rate is suitable for practical applications on Internet of Things, virtual reality, assistive devices, and human-computer interaction applications. This study developed the system based on the gaze point detection for achieving eye tracking results. The fixation points can be used in various ways to achieve eye tracking. In our future works, we will integrate the fixation point clustering-based techniques [34,35] to achieve higher feasibility and reliability on wearable eye tracking.

## Figures and Tables

**Table 1:** Computer details.

Type	Item	Specification
Hardware	CPU	Intel Core i5 1.8 GHz
	RAM	4 GB
Software	OS	Windows 10 Home Edition
	Developing language	Node.js/JavaScript/Visual Studio 2015/C++
	Library	OpenCV 3.10

**Table 2:** Client details.

Type	Item	Specification
Hardware	Model	HTC Desire Eye
	CPU	Qualcomm Snapdragon 801 2.3 GHz
	Screen size	5.2 miles
	Screen resolution	1920 × 1080 pixels
	RAM	4 GB
Software	OS	Android 6.0.1
	Developing language	Android/Java

**Table 3:** Component name and price.

Item Name	Price
Head-mounted device	$49
Mobile phone	$200
Camera	$30
Infrared light emitting element	$4
Else electronic components	$1
3D print material	$1
Total	$285

**Table 4:** Comparative product names and prices.

Product Name	Price
Pupil Labs VR/AR for HTC Vive	About USD$1550 (€1400)
FOVE	$599
This study	$285

**Table 5:** Number of samples.

	Samples	P1	P2	P3	P4	P5	P6	P7	P8	P9
User1	516	58	56	56	57	60	57	58	58	56
User2	440	49	50	48	48	50	49	50	49	47
User3	440	50	49	50	49	49	48	47	50	48
User4	441	50	50	50	48	49	49	47	51	47

**Table 6:** Eye detection result.

	Samples	TP	TN	FP	FN	Accuracy	Precision	Recall
User1	516	512	0	0	4	99.22%	100.00%	99.22%
User2	440	407	4	1	28	93.41%	99.75%	93.56%
User3	440	438	0	0	2	99.55%	100.00%	99.55%
User4	441	440	0	0	1	99.77%	100.00%	99.77%
Average						97.99%	99.94%	98.03%
Lee [18]						98.91%	98.87%	97.84%
Jen [16]						96.86%	98.71%	96.57%
Dobeš [17]						94.43%	98.98%	93.88%

**Table 7:** Sight detection result.

	Frames	TP	TN	FP	FN	Accuracy	Precision	Recall
User1	516	476	0	0	40	92.25%	100.00%	92.25%
User2	440	401	5	0	34	92.27%	100.00%	92.18%
User3	440	435	0	0	5	98.86%	100.00%	98.86%
User4	441	437	0	0	4	99.09%	100.00%	99.09%
Average						95.62%	100.00%	95.60%

**Table 8:** Fixation error.

User		Point 1	Point 2	Point 3	Point 4	Point 5	Point 6	Point 7	Point 8	Point 9	Avg
User1	Accuracy	7.72	6.15	4.04	1.10	0.85	4.30	2.75	1.62	1.90	3.38
	Precision	0.70	0.60	0.40	0.60	0.24	0.76	0.29	0.28	0.40	0.47
User2	Accuracy	5.23	1.78	1.82	3.61	0.86	1.34	1.62	8.51	2.73	3.06
	Precision	6.52	0.79	0.93	3.56	0.26	0.51	0.20	5.82	0.37	2.11
User3	Accuracy	10.13	8.30	2.02	1.86	2.42	2.20	11.16	2.94	2.98	4.89
	Precision	10.57	4.00	0.44	0.54	0.80	0.88	4.09	0.39	0.40	2.46
User4	Accuracy	5.71	2.05	3.40	2.61	0.94	0.80	4.91	1.54	1.89	2.65
	Precision	0.57	1.51	0.19	0.31	0.27	0.22	0.22	0.17	0.72	0.46
Avg	Accuracy	7.20	4.57	2.82	2.30	1.27	2.16	5.11	3.65	2.38	3.50
	Precision	4.59	1.73	0.49	1.25	0.39	0.59	1.2	1.67	0.47	1.38

**Table 9:** Surveys of users.

Question	User1	User2	User3	User4	Average
Ease of use	5	4	4	4	4.25
Easy to navigate	5	3	5	3	4
Adaptively useful	4	5	4	5	4.5
Sufficiency	4	4	3	4	3.75
Enjoyment	4	5	5	5	4.75
Useful	5	4	4	5	4.5
Average	4.5	4.25	4.25	4.33	
